# 

**Published:** 1899-06

**Authors:** 


					' * JUNE, 1899.
Vol. XVII. ^Q>4.
the Ry
s. c. o
BRISTOL
MEDICO-CHIRUlR^jg^r^
I SURGEON GEHEf^-cV-
JOURNAL
15'-1839
S (Sluarterle Journal of tbe /ifceDlcal Sciences for tbe HClest
of England an& Soutb Mates?
PUBLISHED UNDER THE AUSPICES OF
THE BRISTOL MEDICO-CHIRURGICAL SOCIETT.
J
"Scire est nescire, nisi it> mc
Scire alius sciret."
Editor: R. SHINGLETON SMITH, M.D., B.Sc.
WITH WHOM ARE ASSOCIATED
J. MICHELL CLARKE, M.A., M.D., W. H, HARSANT,
B. M. H. ROGERS, B.A., M.D., B.Ch., and JAMES SWAIN, M.S., M.D.
Assistant-Editor: L. M. GRIFFITHS.
Editorial Secretary: JAMES TAYLOR.
BRISTOL: J. W. ARROWSMITH.
London : J. & A. Churchill, 7 Great Marlborough Street.
r- Price One Shilling and Sixpence.

				

